# Studying Mucin-Derived *O*‑Glycopeptides with Gas-Phase FRET

**DOI:** 10.1021/acs.analchem.6c01957

**Published:** 2026-08-21

**Authors:** Kim Greis, Arseniy Galashov, Lyna Bourehil, Ekaterina Kazakova, Oliver Seitz, Renato Zenobi

**Affiliations:** † Laboratory of Organic Chemistry, Department of Chemistry and Applied Biosciences, 27219 ETH Zürich, 8093 Zürich, Switzerland; ‡ Department of Chemistry, 9373 Humboldt-Universität zu Berlin, 12489 Berlin, Germany

## Abstract

At least 50% of human proteins are glycosylated, however, it is not fully understood how glycosylation affects glycopeptide structures. For the highly *O*-glycosylated tandem repeats of the gel-forming mucin MUC5AC, it has been shown that glycoclustering leads to significant stiffening of the peptide backbone in solution. Here, the influence of *O*-glycosylation on the gas-phase structures of synthetic 26-residue MUC5AC-derived model peptides containing either zero or six GalNAc residues is investigated using gas-phase Förster resonance energy transfer (FRET) and ion mobility-mass spectrometry. The results reveal that *O*-glycosylation can induce pronounced compaction after desolvation, in contrast to the glycosylation-induced stiffening in solution. The extent of the compaction is charge state-dependent and likely related to intramolecular solvation of the glycan residues. Additionally, the results show that glycopeptides with Thr-glycosylation are either less compact or of similar size than those with Ser-glycosylation. These results highlight the importance of hydration for stabilizing glycopeptide conformations and show that glycosylation-dependent structural changes should be considered when interpreting gas-phase measurements of glycopeptides.

## Graphical abstract

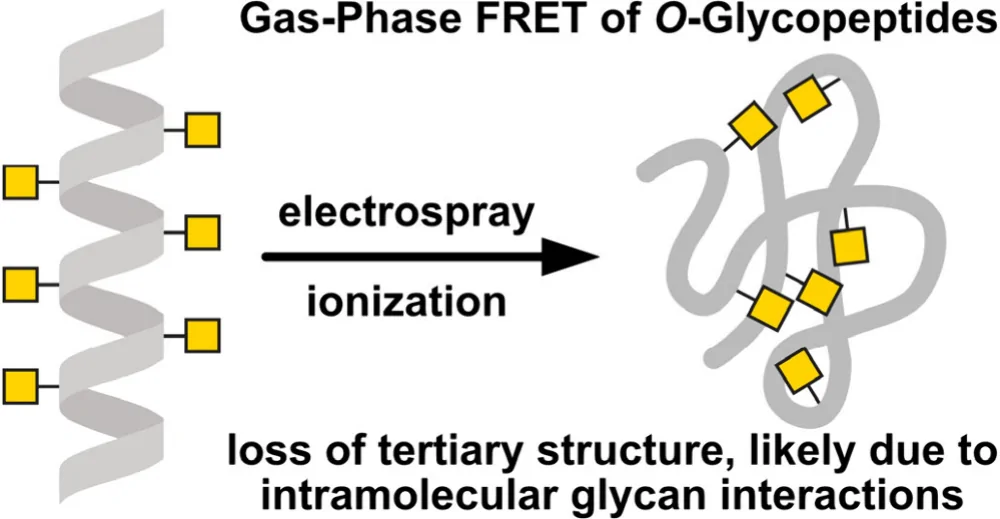

Glycosylation is one of the most widespread and structurally diverse protein modifications. Glycans contribute to molecular recognition processes, including cell–cell communication and host–pathogen interactions, as well as to the folding, stability, and conformational properties of proteins and peptides.^1^ Their structural diversity and site-specific microheterogeneity complicate analytical characterization.^2–4^ Historically, glycans have therefore often been enzymatically or chemically released to simplify analysis.^5^ While this approach facilitates characterization, it can sacrifice information, motivating the direct analysis of intact glycopeptides.

Mucins are heavily *O*-glycosylated proteins that form hydrated mucus gels, which protect epithelial surfaces and facilitate mucus clearance from the airways.^6^ MUC5AC is one of the principal gel-forming mucins of airway mucus and contains tandem-repeat regions rich in proline, threonine, and serine residues, providing dense clusters of *O*-glycosylation sites.^7^ These glycans contribute to the extended, bottlebrush-like architecture characteristic of mucins.^8^ In aqueous solution, GalNAcylation of MUC5AC-derived tandem-repeat peptides stiffens the peptide backbone and favors more extended conformations.^9–12^

Previous mass spectrometry (MS) studies on glycopeptides have focused on determining glycan composition, glycosylation sites, and peptide sequences using tandem-MS.^13–15^ Ion mobility-mass spectrometry (IM-MS) provides complementary information about the structures of intact glycopeptide ions in the gas phase. It was shown that *O*-glycopeptide isomers can be separated using IM-MS.^16–18^ It was further demonstrated that glycopeptide collision cross section (CCS) values depend on glycan composition and charge state.^19–21^ A CCS represents a rotationally averaged measure of the overall size and shape of an ion in a defined drift gas.^22,23^

Native mass spectrometry has become an established tool for characterizing intact biomolecules and biomolecular assemblies, as gentle electrospray conditions can preserve noncovalent interactions and substantial aspects of the solution-phase structure.^24–26^ Direct evidence was provided by experiments showing that tobacco mosaic virus remains infectious after electrospray ionization, ion filtering, and deposition,^27^ as well as by recent cryo-EM analysis of soft-landed β-galactosidase, which revealed retention of large parts of its secondary and tertiary structure.^28^ At the same time, the β-galactosidase study identified dehydration-induced subunit reorientation and compaction.^28^ Another study has shown that enzymes can remain catalytically active inside a mass spectrometer.^29^ While the preservation of native-like structures has been studied extensively for proteins and protein assemblies, the influence of glycosylation on gas-phase structures remains less well understood.

In this study, the effect of *O*-glycosylation on the structure of synthetic MUC5AC-derived model peptides with the sequence Ac-KAPT­TSTT­SAPT­TSTT­SAPT­TSTT­SC-NH_2_ was studied in the gas phase and compared to solution-phase data. Specifically, nonglycosylated 26-amino acid residue peptides and their counterparts carrying six defined *O*-linked GalNAc residues were studied. The MUC5AC-derived APTTSTTS tandem repeat provides a biologically relevant, glycosylation-site-rich model system for investigating the structural effects of clustered *O*-glycosylation. A suitable gas-phase method to study the structure of biomolecules is gas-phase Förster resonance energy transfer (FRET).^30,31^ In systems labeled with a fluorescence donor and a quencher, the extent of fluorescence quenching is strongly distance dependent. Hence, FRET enables the estimation of relative distances between the donor and quencher labels (Scheme 1). Absolute distances can also be determined if the Förster distance *R*_0_ is known. Previous studies have approximated gas-phase *R*_0_ using the value in aqueous buffered solutions.^32–34^ To avoid errors when making such approximations, the current study mainly relies on the relative comparison of quenched lifetimes. Gas-phase FRET is particularly suitable for assessing conformational differences between nonglycosylated and glycosylated peptides, because it probes changes in the distance between the terminal labels. Complementary IM-MS experiments provide CCSs of the (glyco)­peptides. In contrast to the fluorescence lifetimes obtained from gas-phase FRET, an increase in CCS does not necessarily correspond to an increase in distance between the termini of a peptide.

**Scheme 1:**
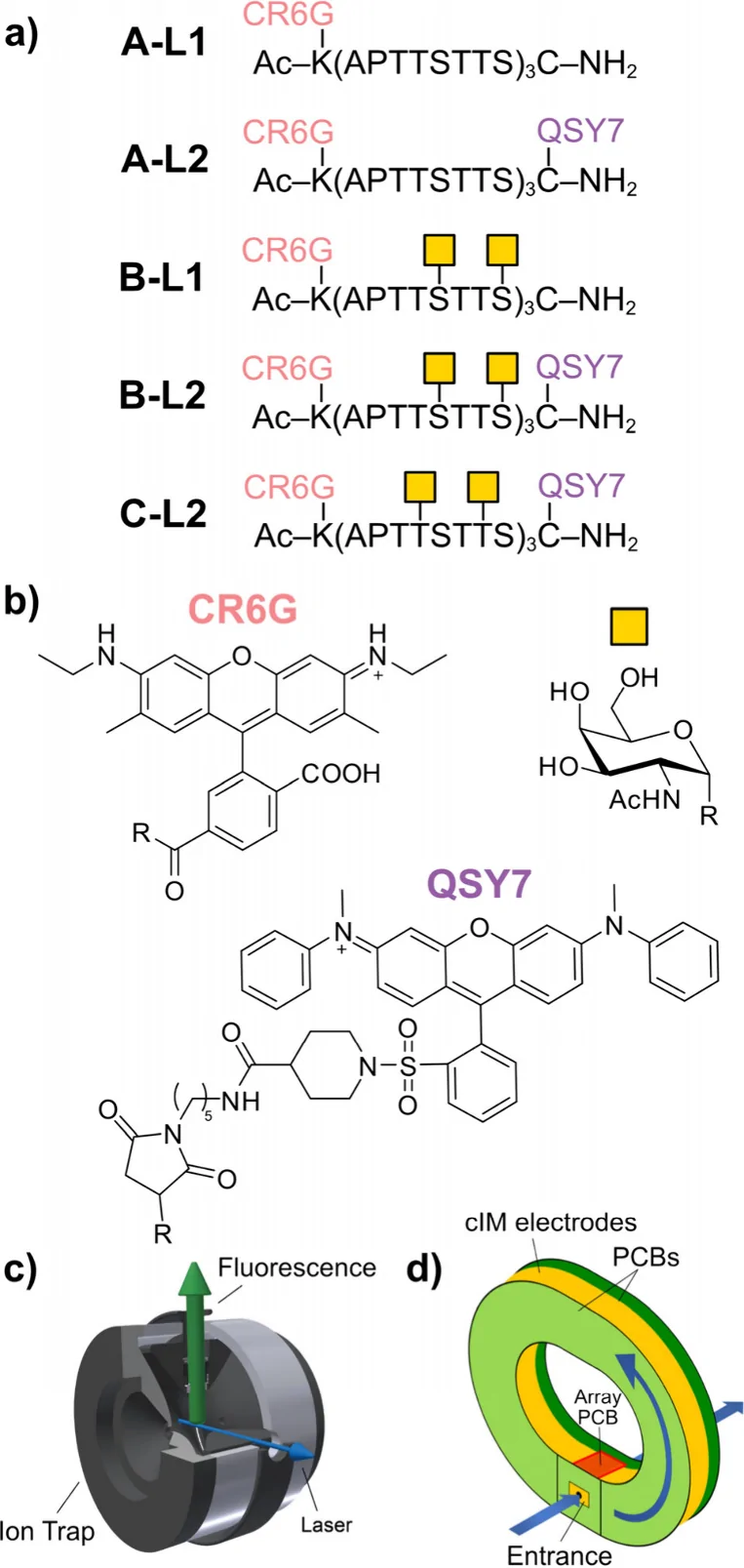
(a) Sequences of (Glyco)­peptides Studied in This Work; (b) Molecular Structures of Fluorescence Donor Carboxyrhodamine 6G (CR6G), Quencher (QSY7), and Post-Translational Modifications by *N*-Acetylgalactosamine (GalNAc, yellow squares); (b) Schematic overview of the cyclic IMS cell used in this study; (c) Schematic Overview of a 3D Ion Trap Interfaced with a Laser Beam for Gas-Phase FRET Studies^a^

a Figure adapted with permission from ref 34. Copyright 2025 American Chemical Society. Figure adapted with permission from ref 40. Copyright 2019 American Chemical Society.

## Experimental Section

### Synthesis

The (glyco)­peptide sequences Ac-K­(APTTSTTS)_3_C-NH_2_, comprising three MUC5AC tandem repeats with the sequence APTTSTTS, were synthesized using solid-phase peptide synthesis with regular and glycosylated (GalNAc, Scheme 1) amino acid building blocks. Three 26-residue peptide variants bearing either zero or six GalNAc residues were synthesized: **A** Ac-K­(APTTSTTS)_3_C-NH_2_, **B** Ac-K­(APTT**S**TT**S**)_3_C-NH_2_, and **C** Ac-K­(APT**T**ST**T**S)_3_C-NH_2_ (with bold amino acids being glycosylated). The lysine residues were labeled with the fluorophore 5(6)-carboxyrhodamine 6G (CR6G) via an HATU/Oxyma/DIPEA mediated reaction on-resin, leading to the singly labeled (glyco)­peptides (**A-L1** and **B-L1**). After peptide cleavage and purification, the cysteine residues were labeled with the nonfluorescent quencher QSY7 via a maleimide reaction in-solution, leading to doubly labeled (glyco)­peptides (**A-L2**, **B-L2**, and **C-L2**). The products obtained were purified using HPLC and lyophilized (Figure S1). Finally, they were dissolved in water to afford 26 μM (**A-L1**), 41 μM (**A-L2**), 61 μM (**B-L1**), 77 μM (**B-L2**), and 131 μM (**C-L2**) stock solutions. Aliquots of the stock solutions were kept at −80 °C, where they remain stable for months. A detailed description of the synthesis procedure of fluorescent (glyco)­peptides can be found in the SI.^35^

### Mass Spectrometry and Ion Mobility-Mass Spectrometry

The (glyco)­peptides were diluted to 5 μM in 50 mM aqueous ammonium acetate (AA) added as a volatile additive for MS experiments. The (glyco)­peptides were ionized via positive ion mode nanoelectrospray ionization (nESI) using borosilicate glass emitters (Science Products GmbH, Hofheim, Germany) that were pulled to a tip using a micropipette puller (P-1000, Sutter Instrument, Novato, CA, USA). High voltage (1.0–1.5 kV) was applied to the emitter containing the sample through a platinum wire. Mass spectra were acquired on a Synapt G2S mass spectrometer (Waters, Wilmslow, Manchester, UK).

IM-MS experiments were conducted on a Waters Select Series Cyclic IMS mass spectrometer (Milford, MA, USA) in positive ion mode (Scheme 1d). The nESI setup is identical to that used for acquiring mass spectra. To obtain a higher ion signal, increased (glyco)­peptide concentrations of 20 μM with 50 mM AA were used. To have access to lower charge states (2+), experiments were repeated in 80 mM AA and 20 mM triethylammonium acetate (TEAA). The drift times were recorded in nitrogen. Melittin, ubiquitin, and cytochrome c were selected as calibrants to convert arrival time distributions (ATDs) into CCS distributions^36–39^ (Supporting Information, Figure S6 and Table S4). Three IMS wave heights (24, 25, and 26 V) were employed to assess the effect of calibration conditions, at a constant wave velocity of 350 m s^–1^.^36^ Then, ATDs of the studied peptides were acquired at the selected wave heights and converted into CCS distributions. All data were extracted using MassLynx v4.2 and DriftScope v3.0 (Waters, Manchester, UK).

### Gas-Phase FRET

Gas-phase FRET experiments were conducted on a custom-built setup, which was described previously.^32,41^ Briefly, (glyco)­peptides were ionized *via* positive ion mode nESI, as described in the mass spectrometry section, from 20 μM solutions containing AA (50 mM for the [M + 3H]^3+^ and [M + 3H]^4+^ charge states or 10 mM for the [M + 4H]^4+^ and [M + 4H]^5+^ charge states). The ions were *m*/*z*-selected and stored in a LCQ quadrupole ion trap mass spectrometer (Thermo Fisher, Waltham, MA, USA) interfaced with a laser and a photon detector. A 460 nm laser beam with a repetition rate of 80 MHz was generated using a Ti:sapphire fs laser (MaiTai, Spectra-Physics, USA) with frequency doubling by a second harmonic generator (UHG, Spectra-Physics, USA). A pulse picker (350–105-UV, Conoptics, Danbury, CT, USA) was used to reduce the repetition rate to 26.7 MHz. The laser beam (7–10 mW) is guided by optical mirrors into the 3D ion trap (Scheme 1c), where it overlaps with the ion cloud for 1 s. For each cycle, the trap is refilled with newly generated ions. The fluorescence of the activated ions is collected orthogonally to the laser beam, via a set of lenses and mirrors guiding a fraction of the emitted fluorescence to a single-photon avalanche diode (SPAD, Picoquant GmbH, Berlin, Germany) for observing the emitted fluorescence as a function of time. Fluorescence lifetimes were obtained from fitted fluorescence decay curves *via* a custom Python script.^42^

### Solution-Phase FRET

Solution-phase fluorescence studies were conducted using the same laser setup and a second SPAD detector, identical to the one used for gas-phase studies. The laser beam (480 nm, 8–650 μW) is guided to a cuvette containing an aqueous 250 nM (glyco)­peptide solution in pure water, 50 mM AA, or 100 mM phosphate buffer (Tables S2 and S3 and Figure S4).

### Computational Section

(Glyco)­peptide model structures APTTSTTS-NH_2_ were generated and their conformational space sampled using CREST^43^ with the GFN2-xTB^44^ method. 2+ and 3+ charge states were generated using the protonate tool implemented in CREST with the GFN2-xTB method. The ten lowest-energy conformers/protomers of every sampling step were optimized in ORCA 6.0.1 ^45^ using the DFT method B97–3c.^46^ Atomic charges of the optimized geometries were calculated with the CHELPG^47^ method in ORCA at the same level of theory. The tool hpccs^48^ was used to generate computed CCSs with the trajectory method (Table S5) using the optimized geometries and their atomic charges (drift gas: nitrogen).

## Results and Discussion

Synthesis and labeling of (glyco)­peptides with the sequence Ac-K­(APTTSTTS)_3_C-NH_2_ proceeded through established protocols,^9^ which are described further in the Experimental Section and in the SI. PTS (proline, threonine, serine) domains such as the APTTSTTS tandem repeats of MUC5AC are glycan-rich domains, forming bottlebrush structures that trap water, leading to the formation of gels.^8,49–51^ The terminal amino acid residues K and C were chosen because they can be independently labeled with fluorescent and quencher dyes CR6G and QSY7, respectively.

When ionized by positive nESI from aqueous AA solutions, (glyco)­peptides are primarily detected as protonated species (Figures 1 and S2). Using AA as a volatile additive increases the pH of the analyte solution to 6.8 (from 5.3–5.8 in pure water). While the pH is expected to decrease during ionization in positive ion mode, followed by desolvation, to around 4.8 in AA, it prevents the strong acidification, which occurs during ionization from pure water.^52^ Nevertheless, AA remains a useful additive in native MS and its effectiveness for retaining native-like structures during ionization is well documented for proteins.^24^ Likewise, it can be expected that also for (glyco)­peptides, AA ensures *native* ionization conditions, and promotes the formation of protonated species. Without adding AA, the propensity for sodium- and potassium-adduct formation increases significantly. The singly labeled species (**L1**) ionize as [M + *x*H]^*x*+^ ions, whereas doubly labeled species (**L2**) ionize as [M + (*x* – 1)­H]^*x*+^ ions. This is due to the intrinsic charge of the QSY7 quencher, leading to a higher charge than the number of protons added during ionization.

**Figure 1:**
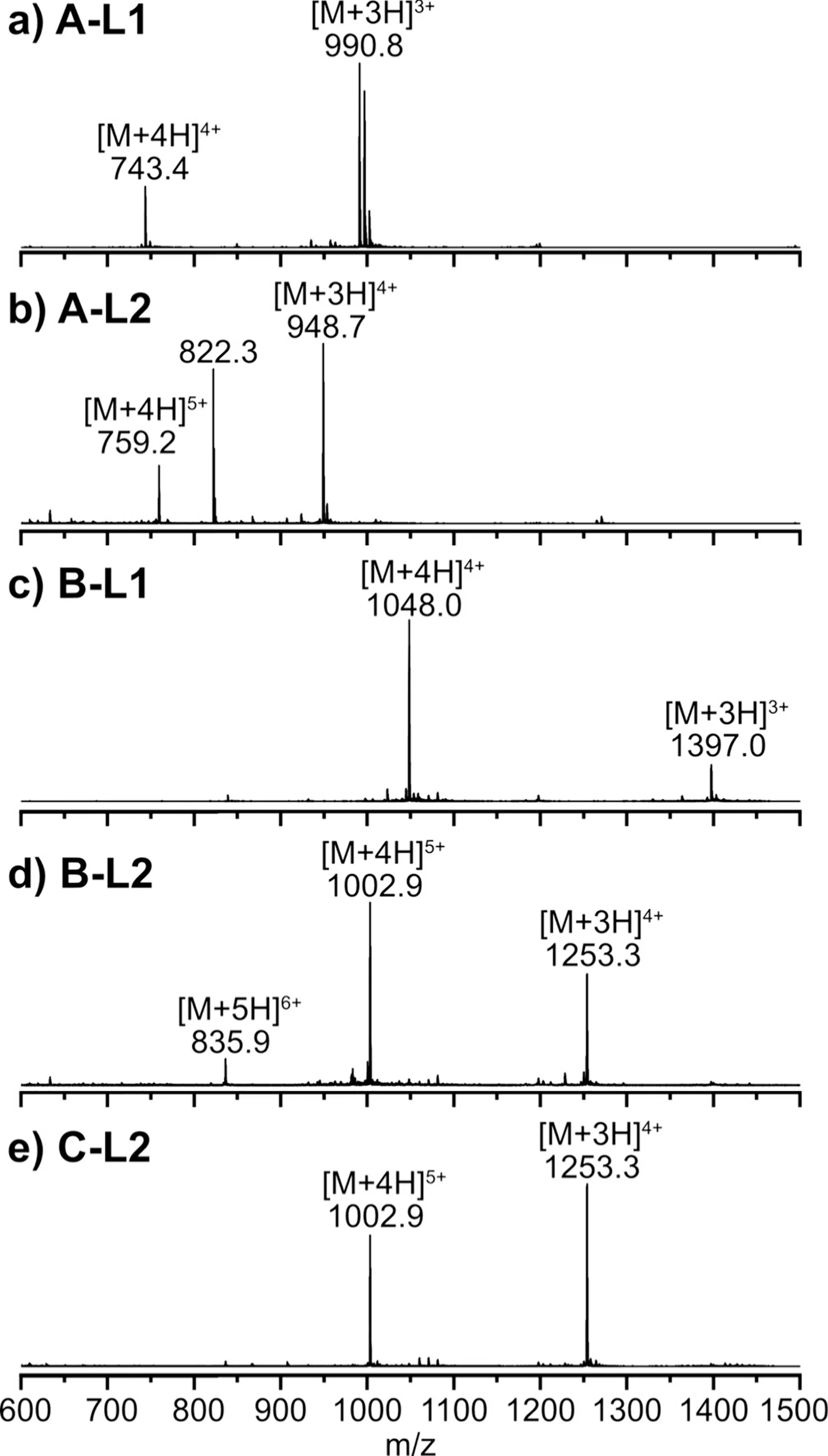
ESI-(+)-mass spectra of the (glyco)­peptides (a) **A-L1**, (b) **A-L2**, (c) **B-L1**, (d) **B-L2**, and (e) **C-L2**. When ionized from aqueous solution containing ammonium acetate as a volatile additive, mainly protonated peptides are obtained. The signal at *m*/*z* 822.3 in (b) is due to an impurity consisting of the unreacted QSY7 quencher labeling reagent. **A-L1** and **B-L1** form [M + *x*H]^*x*+^ ions, whereas **A-L2**, **B-L2**, and **C-L2** form [M + (*x* – 1)­H]^*x*+^ ions due to the intrinsic charge of the QSY7 dye.

Gas-phase fluorescence experiments were conducted on the charge states [M + 3H]^3+^ and [M + 3H]^4+^ for the (glyco)­peptides **A-L1**/**B-L1** and **A-L2**/**B-L2**, respectively (Figure 2). The singly labeled (glyco)­peptides **A-L1** and **B-L1** in their [M + 3H]^3+^ charge state exhibit fitted fluorescence lifetimes of 6.28 and 6.96 ns, respectively. Additionally, **B-L1** was also probed in the [M + 4H]^4+^ charge state (Figure S3), where its fluorescence lifetime decreases to 6.25 ns. These (glyco)­peptides only carry a fluorescence donor and not a quencher, which is why this lifetime corresponds to the intrinsic lifetime of the fluorescent CR6G dye in each system. It is known from previous studies that unquenched gas-phase fluorescence lifetimes of species labeled with CR6G can span 5.5 to 7.5 ns depending on the molecule it is labeled to, the labeling position, and the charge state.^53–55^ Certain amino acid residues such as tryptophan, for example, can induce partial quenching of the fluorescence. A recent study emphasized the influence of nearby charges on fluorescence quenching.^56^ Additionally, nonquenched lifetimes of a dye are usually longer in the gas phase than in the solution phase (Tables S1–S3) because interactions of the fluorophore with the peptide backbone increase its rigidity, limiting nonradiative vibrational or rotational decay.

**Figure 2:**
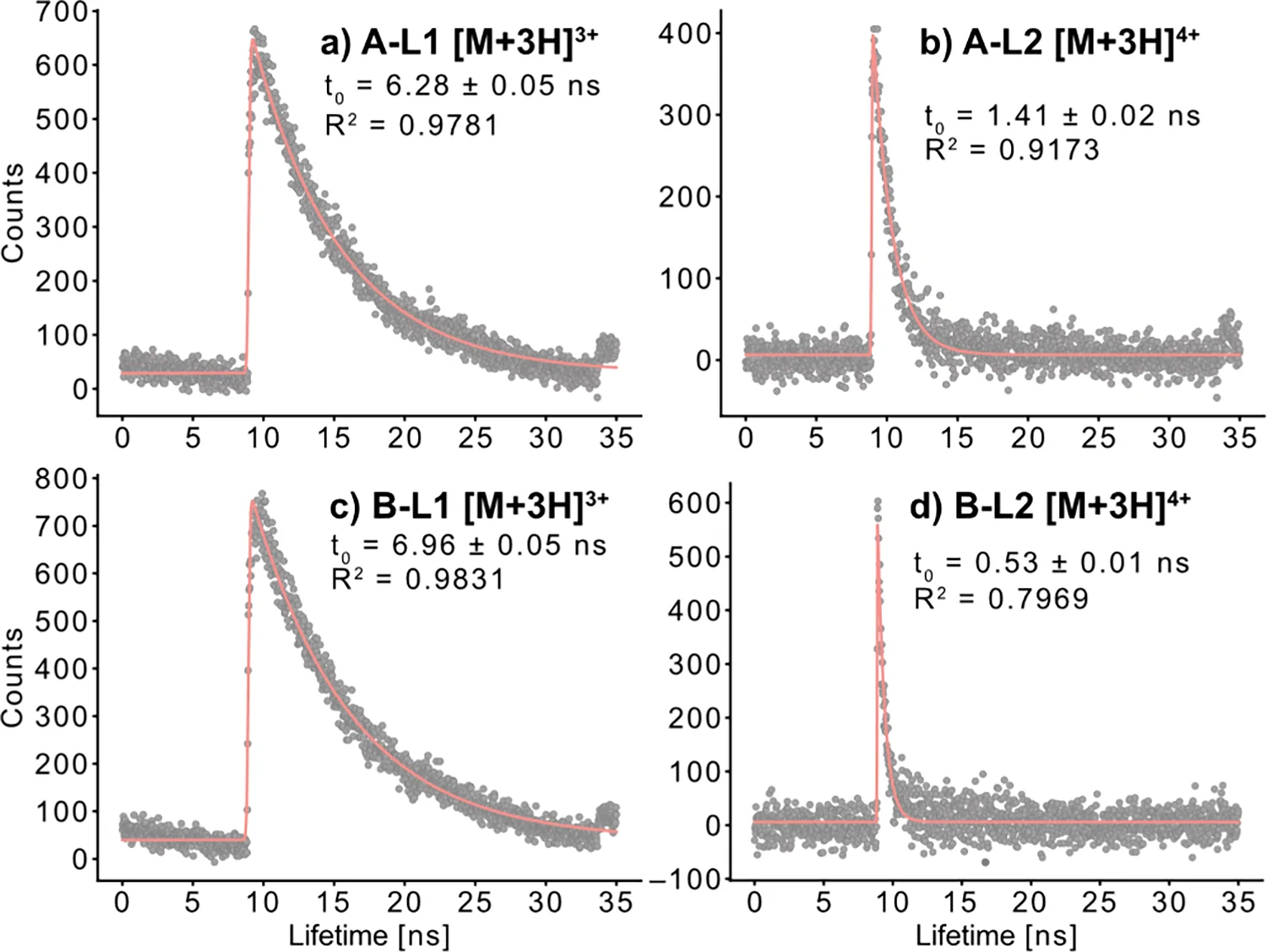
Gas-phase fluorescence decay curves of the (glyco)­peptides (a) **A-L1**, (b) **A-L2**, (c) **B-L1**, and (d) **B-L2** in the charge states [M + 3H]^3+^ and [M + 3H]^4+^. Gray circles represent single data points and the fit through these data points is represented by a red curve. The fluorescence lifetime is obtained through the fit. The error is the statistical error of the fit. The quenched fluorescence lifetime of nonglycosylated **A-L2** is longer than that of glycosylated **B-L2**, indicating that glycopeptides collapse to a higher degree than nonglycosylated peptides.

The gas-phase fluorescence lifetimes of **A-L2** (1.41 ns) and **B-L2** (0.53 ns) in their [M + 3H]^4+^ charge state, on the other hand, indicate significant quenching by the QSY7 label. The fluorescence lifetime of **B-L2** is shorter than that of **A-L2**. A shorter fluorescence lifetime indicates stronger quenching, which corresponds to higher FRET efficiency (Tables 1, S1, and S3) and thus a shorter distance. This result is intriguing, because this means that the distance between the two labels, and therefore the distance between the termini of the peptide sequence, is shorter in the glycopeptide **B-L2** than in its nonglycosylated counterpart **A-L2**. We expected an increase in distance between the termini upon glycosylation, based on reported solution-phase experiments using pulsed electron–electron double resonance spectroscopy (PELDOR) on similar systems.^9^ Motivated by these results, we investigated whether this phenomenon is exclusive to the gas phase or is also true in the solution phase. Therefore, the labeled (glyco)­peptides were also studied using solution-phase FRET (Figure S4 and Tables 1, S2, and S3). The solution-phase results show that the nonquenched lifetimes of **A-L1** and **B-L1** are significantly lower than in the gas phase and almost identical with 4.44 and 4.45 ns, respectively. The trend of the quenched lifetimes of **A-L2** and **B-L2** is with 2.65 and 3.50 ns in agreement with the reported PELDOR experiments.^9^ The results confirm that in the aqueous phase glycosylation of a peptide backbone leads to stiffening.

**Table 1:** Comparison of Gas- And Solution-Phase FRET Efficiencies^a^

	gas phase	solution phase
FRET efficiency [%]	[M + 3H]^4+^	H_2_O, 50 mM AA
**A-L2**	77.5	40.3
**B-L2**	92.4	21.3
**C-L2** ^a^	93.1	2.7

a The efficiencies indicate that, upon glycosylation, the peptides adopt more compact conformations in the gas phase for the 4+ charge state, whereas stiffening of the same peptide backbone occurs in the solution phase. Standard deviations are indicated in the SI (Tables S1 and S3).

b These values are approximated by using the unquenched lifetime of **B-L1**.

Hence, in the gas phase the decrease in distance between the terminal residues of the glycopeptide **B-L2** compared to its nonglycosylated counterpart **A-L2** indicates that the propensity of the investigated glycopeptides to retain their native structure upon nESI is lower than that of peptides. While there is a growing body of literature supporting that peptides and proteins retain large aspects of their native solution-phase structure upon ionization,^27,28,57–60^ our results show that for glycopeptides this is not necessarily the case. The decreased propensity of the investigated glycopeptides to retain their overall structure can be explained as follows. In aqueous solution the three-dimensional structure of a protein is mainly driven by the hydrophobic effect as well as interactions between polar residues, e.g., hydrogen bonds or salt bridges. These interactions are stronger than the interactions of the involved residues with solvent water and can be retained upon gentle ionization on the time scale of an MS experiment. Glycan residues, on the other hand, are highly polar and rely more strongly on stabilization by water^61^ or other highly polar moieties, such as neighboring glycan residues. Upon ionization and transfer to the gas phase, the glycan moieties interact with the peptide backbone instead of being stabilized through solvation. Hence, the surrounding amino acid residues and the peptide backbone will fold around the glycans, effectively leading to the collapse of the glycopeptide’s native secondary and tertiary structures.

To further confirm the findings, the investigated (glyco)­peptides were analyzed with IM-MS to derive CCSs (Tables 2 and S4 and Figure S5). Due to the correlation between mass and mobility, a CCS is expected to increase when bulky post-translational modifications, such as glycans, are added to a peptide.^19,21^ Therefore, unlike FRET lifetimes, a CCS does not necessarily reflect the distance between the peptide’s termini.

**Table 2:** Collision Cross Sections of (Glyco)­peptides Determined *via* Cyclic Travelling Wave Ion Mobility Spectrometry (TWIMS) Using N_2_ as Drift Gas^a^

CCS [Å^2^]	2+^a^	3+	4+	5+
**A-L1**	608	685	901	
**B-L1**	727	760	943	
**A-L2**	711	770	1041	1165^a^
**B-L2**	496	854	985	1239
**C-L2**	629	848	989	1260

a The 5+ charge state of the (glyco)­peptides **A-L1** and **B-L1** could not be generated.

b These CCSs were determined for only one wave height.

Standard deviations are indicated in the SI (Table S4).

Unexpectedly, for the 4+ charge state, which is the one that was also investigated with gas-phase FRET, the CCS of the nonglycosylated peptide **A-L2** (3795 Da) exceeds that of the glycopeptides **B-L2** and **C-L2** (5013 Da) by 56 and 52 Å^2^, respectively. These results confirm the gas-phase FRET findings in Figure 2 showing that for these species a compaction can be observed when the investigated peptide carries six GalNAc residues, contrasting the stiffening that can be observed in solution upon glycosylation.^9^ A similar observation can be made for the 2+ charge state, where the CCSs decrease by 215 and 82 Å^2^ when going from **A-L2** to **B-L2** and **C-L2**, respectively. For the other charge states and the singly labeled (glyco)­peptides **A-L1** and **B-L1**, the CCS increases upon glycosylation by 42–119 Å^2^. The average change in CCS when going from nonglycosylated to glycosylated is +15 Å^2^. Generally, CCSs increase upon glycosylation for the singly labeled peptides (**A-L1** and **B-L1**), whereas the behavior of the doubly labeled peptides (**A-L2**, **B-L2**, and **C-L2**) is charge state-dependent. This difference indicates that the fluorophore labels can influence the gas-phase conformational behavior of the studied (glyco)­peptides.

To assess the expected increase in CCS upon glycosylation, the conformational spaces of model (glyco)­peptides with the sequence APTTSTTS-NH_2_ (one tandem repeat) were computationally sampled and the CCS of representative structures determined. The model sequences carry no, one, or two GalNAc residues. As in the experimentally studied peptides, GalNAc moieties were placed either on the serine residues or on the second and fourth threonine residues. The results (Table S5) indicate that the CCS increases by 40 ± 15 Å^2^ per GalNAc residue. The experimentally studied glycopeptides carry six GalNAc residues and therefore an increase in CCS of approximately 240 Å^2^ would be expected. As the experimental CCSs either decrease upon glycosylation or show an increase significantly lower than what is expected based on the computational model, it is believed that to attain such low CCS values, the glycopeptides likely undergo glycosylation-induced compaction in the gas phase. A previous study on *N*-glycopeptides derived from digested glycoproteins determined incremental increases of 11–19 ± 5 Å^2^ per Gal and 13–15 ± 5 Å^2^ per GlcNAc residue.^21^ The studied peptide sequences were typically shorter, with less abundant but more complex glycan sites, than for the MUC5AC-derived model peptides studied in this work. In our IM-MS experiments, we see decreases down to −36 Å^2^ and increases up to 20 Å^2^ per GalNAc residue. Overall, our results are in line with recent studies showing that adding peptide posttranslational modifications can lead to a decrease in CCS despite the increase in mass, which is mainly attributed to intramolecular charge solvation.^19,62^

In addition, the influence of the glycan position on the glycopeptide structures was investigated. In **B-L2**, all serines are glycosylated, whereas in **C-L2**, every second threonine is glycosylated. From solution-phase experiments,^9^ it is known that Thr-glycosylation leads to more elongated glycopeptides than Ser-glycosylation in aqueous solutions (Figure S4). The fluorescence lifetimes (Figure 3) indicate that for the [M + 3H]^4+^ charge state, the distance between the termini is identical within experimental error. For the [M + 4H]^5+^ charge state, on the other hand, the fluorescence lifetime of **C-L2** is 0.52 ns longer than that of **B-L2**, indicating that the interterminal distance in **C-L2** is longer than that in **B-L2**. Hence, the results indicate that in the gas phase the influence of the glycan position is charge-state-dependent and glycosylation of threonine either leads to similar or longer interterminal distances.

**Figure 3:**
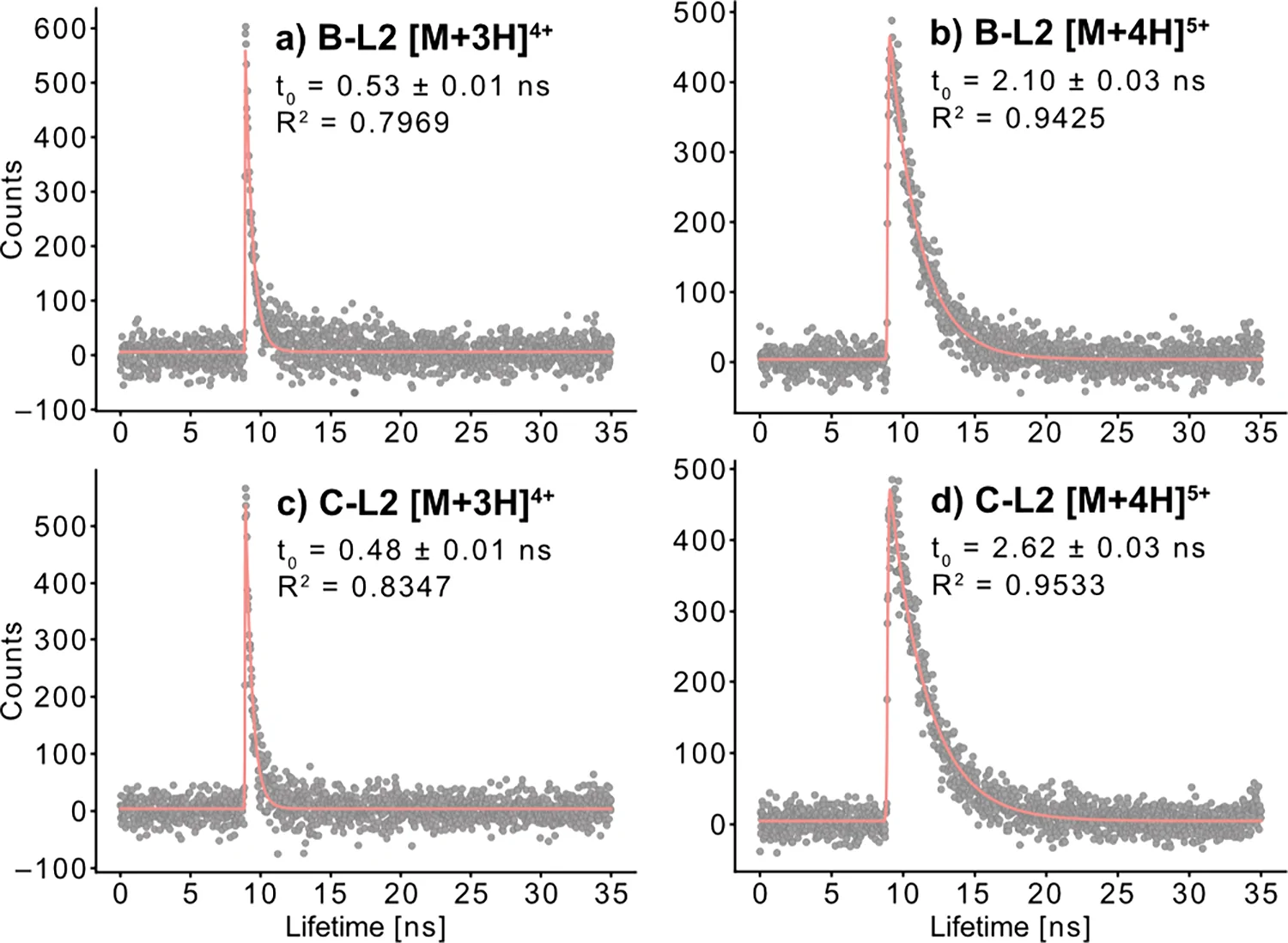
Gas-phase fluorescence decay curves of the glycopeptides **B-L2** in the charge states (a) [M + 3H]^4+^ and (b) [M + 4H]^5+^, as well as **C-L2** in the charge states (c) [M + 3H]^4+^ and (d) [M + 4H]^5+^. Gray circles represent single data points and the fit through these data points is represented by a red curve. The fluorescence lifetime is obtained through the fit. The indicated error is the statistical error of the fit. While the fitted lifetimes appear almost identical for both glycopeptides in the [M + 3H]^4+^ charge state, the data shows that the lifetime of **C-L2** is longer than that of **B-L2** in the [M + 4H]^5+^ charge state, indicating that glycosylation at threonine leads to longer interterminal distances than serine glycosylation.

These results are generally confirmed by IM-MS (Table 2). The CCSs of **C-L2** are larger than those of **B-L2** for the 2+ and 5+ charge states and the same within error for the 4+ and 3+ charge states, respectively. These findings confirm that glycosylation at threonine generally leads to longer or similar-sized structures than glycosylation at serine in the gas phase. This matches the trend in the aqueous phase, as determined by solution-phase FRET (Figure S4) and PELDOR experiments.^9^

The present results provide evidence for charge state-dependent compaction of the 26-residue model glycopeptide cations studied here. How this behavior translates to larger glycopeptides and glycoproteins bearing more extended *N*- or *O*-glycans remains to be established. We hypothesize that desolvation promotes intramolecular contacts between the glycan residues and the peptide or protein surface. For larger glycans, intra- and interglycan interactions may compete with glycan–backbone collapse. As native MS studies have shown, proteins can retain large aspects of their solution-phase structure and should therefore be more stable, even as the glycans collapse onto their surface.^28^ Smaller peptides, like the ones in this study, are expected to be more prone to change their structure upon collapse of the glycans onto the backbone.

Different behavior may be expected for acidic glycans in negative-ion mode. Here, the localization of the negative charge of the glycan residues might lead to the formation of ion-dipole interactions.^63^ A recent native IM-MS and surface-induced dissociation study of pentraxin-2 showed that glycosylation can substantially influence the stability of protein complexes.^64^ Terminally sialylated *N*-glycans were found to stabilize pentameric and decameric assemblies. These results indicate that glycan–protein interactions in larger systems can stabilize defined tertiary or quaternary structures.

## Conclusions

In conclusion, this study shows that the conformational behavior of glycopeptides in the gas phase can differ substantially from that observed in the solution. By combining gas-phase FRET, IM-MS, and computational modeling, we demonstrated that MUC5AC-derived model *O*-glycopeptides show pronounced charge-state-dependent compaction after desolvation. This behavior contrasts with the glycosylation-induced stiffening of the same peptide backbone observed in aqueous solution.^9^ This compaction is most likely due to the lack of water in the gas phase, which promotes the intramolecular solvation of the glycan residues.

The positions of the GalNAc residues also influence the gas-phase conformation. Glycosylation at threonine results in a structure that is similar in size to or less compact than the corresponding serine-glycosylated sequence, depending on the charge state. This trend is broadly consistent with the solution-phase behavior while also emphasizing that the gas-phase structures depend on the glycosylation pattern and charge state.

These results show that glycosylation-dependent structural changes should be considered when interpreting gas-phase measurements of glycopeptides. The extent to which the observed compaction applies to other peptide sequences, glycan structures, and larger glycoproteins remains to be established. In this study, gas-phase FRET is applied to glycopeptides for the first time. The results demonstrate that gas-phase FRET provides structural information beyond that accessible by IM-MS alone. While the CCS reflects the overall size and shape of an ion, glycosylation-induced structural changes may be partially masked in the IM-MS measurements. Gas-phase FRET provides a complementary distance-dependent readout providing additional information on conformational changes that can occur upon glycosylation. Future studies involving additional peptide sequences and glycosylation patterns, as well as an investigation of the influence of the fluorophore labels, will be required to determine how broadly the observed compaction extends across gas-phase glycopeptide systems.

## Supplementary Material



## Data Availability

The data that support the findings of this study are openly available in ETH Zurich Research Collection at 10.3929/ethz-c-000796088.

## Notes

The Supporting Information is available free of charge on the ACS Publications Web site. The Supporting Information is available free of charge at https://pubs.acs.org/doi/10.1021/acs.analchem.6c01957.

A detailed description of the synthesis, mass spectra, solution-phase FRET, ion mobility, and computational results (PDF)
