# Supplementary material for: Studying Mucin-Derived O‑Glycopeptides with Gas-Phase FRET
Source: Anal Chem. 2026 Aug 21;98(35):25626–34. doi: 10.1021/acs.analchem.6c01957 (PMC13564040; doi:10.1021/acs.analchem.6c01957)
Supplement: Supplementary file 1 [file ac6c01957_si_001.pdf]

Supporting Information

Studying Mucin-Derived *O*-Glycopeptides with Gas-Phase FRET

Kim Greis,*[a] Arseniy Galashov,[b] Lyna Bourehil,[a] Ekaterina Kazakova,[b] Oliver Seitz,[b] Renato Zenobi*[a]

[a] Laboratory of Organic Chemistry, Department of Chemistry and Applied Biosciences, ETH Zürich, CH-8093 Zürich, Switzerland
[b] Department of Chemistry, Humboldt-Universität zu Berlin, DE-12489 Berlin, Germany

Correspondence to: kim.greis@fulbrightmail.org
zenobi@org.chem.ethz.ch

Table of Contents 2
Solid-Phase (Glyco)Peptide Synthesis 3
Mass Spectrometry 6
Gas-Phase FRET 7
Solution-Phase FRET 9
Ion Mobility–Mass Spectrometry 11
Computational Results 13
References 13

Solid-Phase (Glyco)Peptide Synthesis

Commercially available compounds were used without further purification. TentaGel R RAM resin was purchased from Rapp Polymers. Fmoc-Thr/Ser(αAc_3GalNAc)-OH were prepared according to a published procedure[1]. **Fmoc-SPPS** was performed at 40 µmol scale by using a Biotage Initiator+ Alstra synthesizer (Uppsala, Sweden) as previously published.[2] To minimize the number of HPLC purification steps, the 5(6)-CR6G label was introduced on solid phase. However, this step proved challenging. Coupling yields were low. Furthermore, in an unexpected reaction, the 5(6)-CR6G structure was found to be converted to the rhodamine hydrazide when the resin was treated with hydrazine for removal of *O*-acetyl groups on GalNAc residues. Therefore, *O*-acetyl groups were removed by treating the resin-bound glycopeptide with NaOMe in DMF/MeOH. By-products formed by β-elimination of glycans were separated by HPLC purification applying shallow gradients (*vide infra*), which also allowed the separation of CR6G isomers.

Analytical HPLC-MS measurements were performed by using an Agilent 1290 Infinity II LC System, equipped with a Poroshell 120 EC-C18 column (2.1 x 50 mm, 1.9 µm; heater set to 50 °C) with a binary mixture of A (0.1% TFA, 1% ACN, 98.9% H_2O) and B (0.1% TFA, 1% H_2O, 98.9% ACN) as a mobile phase (flow = 0.5 mL/min) in a linear gradient.

Preparative HPLC purifications were carried out by using an Agilent 1100 Series system equipped with a Nucleodur C18 Gravity column (250 mm x 21 mm, 5 µm) from Macherey-Nagel with a binary mixture of A (0.1% TFA, 1% ACN, 98.9% H_2O) and B (0.1% TFA, 1% H_2O, 98.9% ACN) as a mobile phase (flow = 15 mL/min) in a linear gradient.

Fmoc-removal: The resin was treated with deprotection solution (20% Pyrrolidine in 1,3-Dioxolane (DOL) + 1% Tween-20 (v/v)) for 2x5 min. The resin was washed with 3x2 mL of DOL, each washing was performed for 1 minute.

Coupling of usual amino acids: Fmoc-protected amino acids (6 eq.) were transferred to the resin, followed by DIC and Oxyma (6 eq. each). The coupling mixture contained 120 mM of amino acid and 1% Tween-20 (v/v). Coupling time was extended to 10 min. The resin was washed with 2x2 mL of DOL, each washing was performed for 1 minute.

Coupling of glycoamino acids: Fmoc-protected amino acids (1.5 eq.) were transferred to the resin, followed by DIC and Oxyma (1.5 eq. each). The coupling mixture contained 60 mM of amino acid and 1% Tween-20 (v/v). Coupling time was 10 min. The resin was washed with 2x2 mL of DOL, each washing was performed for 1 minute.

Capping: The resin was treated with DOL:Ac_2O:DIPEA (70:20:10, v/v/v) for 5 min at RT. The resin was washed with 3x2 mL of DOL, each washing was performed for 1 minute.

Labeling with 5(6)-CR6G: Prior to CR6G introduction, the Fmoc group was removed from N^{ε}-lysine as described. Then, 5(6)-CR6G hydrochloride (1.5 eq.), HATU (1.5 eq.), Oxyma (1.5 eq.) and DIPEA (4.5 eq.) were dissolved in DMF and transferred to the resin. The final concentration of 5(6)-CR6G was 63 mM. The reaction proceeded overnight. Afterwards, the resin was washed with DMF, and peptides were subjected to the *TFA cleavage* (for non-glycosylated peptides), or to the *Removal of O-acetyl groups on resin* (for glycopeptides).

Removal of O-acetyl groups on resin: 1 mL of DMF-MeOH solution (85:15), containing freshly prepared NaOMe (6 mM) was added to the resin. After 10 minutes, the mixture was removed and the resin was washed with 5x1 mL of DMF, 5x1 mL of CH_2Cl_2, and 2x1 mL of Et_2O.

TFA cleavage: 20% of the resin obtained after deacetylation was forwarded to acidolysis. The resin was washed with CH_2Cl_2 and dried under vacuum. Then 5 mL of a mixture of TFA:TIS:H_2O:EDT (94:2:2:2,

v/v/v/v) was added to the resin. After 2 h the cleavage cocktail was collected by filtration, the resin was washed once with 5 mL of TFA and the combined filtrates were concentrated under argon flow to 5 mL.

Peptide work-up: To the remaining residue, cold Et_2O (9-fold volume) was added and the suspension was centrifuged (4200 rpm, 5 min). Afterwards the ether phase was decanted. The peptide precipitate was dissolved in water.
The peptides Ac-K^{CR6G}-$(APTTSTTS)_3$-C (**A-L1**), Ac-K^{CR6G}-(APTTŠTTŠ$)_3$-C (**B-L1**), and Ac-K^{CR6G}-(APTŤSTŤS$)_3$-C (**C-L1**) were produced.

Ac-K^{CR6G}-$(APTTSTTS)_3$-C (**A-L1**)
Preparative HPLC 20-25% B in 40 min. Yield: 0.25 mg (83.7 nmol) of major CR6G isomer, 1%. UPLC: t_R = 4.36 min (20-25% B in 10 min); ESI-MS (pos. mode): m/z = 990.8 ($C_{128}H_{199}N_{30}O_{49}S$ $(M+3H)^{3+}$, calcd.: 990.8, 743.4 ($C_{128}H_{200}N_{30}O_{49}S$ $(M+4H)^{4+}$, calcd.: 743.3).

Ac-K^{CR6G}-(APTTŠTTŠ$)_3$-C (**B-L1**)
Preparative HPLC 17-22% B in 40 min. Yield: 0.07 mg (17.48 nmol) of major CR6G isomer, 0.2%. UPLC: t_R = 6.25 min (17-22% B in 10 min); ESI-MS (pos. mode): m/z = 1397.0 ($C_{176}H_{277}N_{36}O_{79}S$ $(M+3H)^{3+}$, calcd.: 1396.9, 1048.0 ($C_{176}H_{278}N_{36}O_{79}S$ $(M+4H)^{4+}$, calcd.: 1048.0)

Ac-K^{CR6G}-(APTŤSTŤS$)_3$-C (**C-L1**)
Preparative HPLC 17-22% B in 40 min. Yield: 0.09 mg (20.68 nmol) of major CR6G isomer, 0.3%. UPLC: t_R = 6.86 min (17-22% B in 10 min); ESI-MS (pos. mode): m/z = 1397.3 ($C_{176}H_{277}N_{36}O_{79}S$ $(M+3H)^{3+}$, calcd.: 1396.9, 1048.6 ($C_{176}H_{278}N_{36}O_{79}S$ $(M+4H)^{4+}$, calcd.: 1048.0)

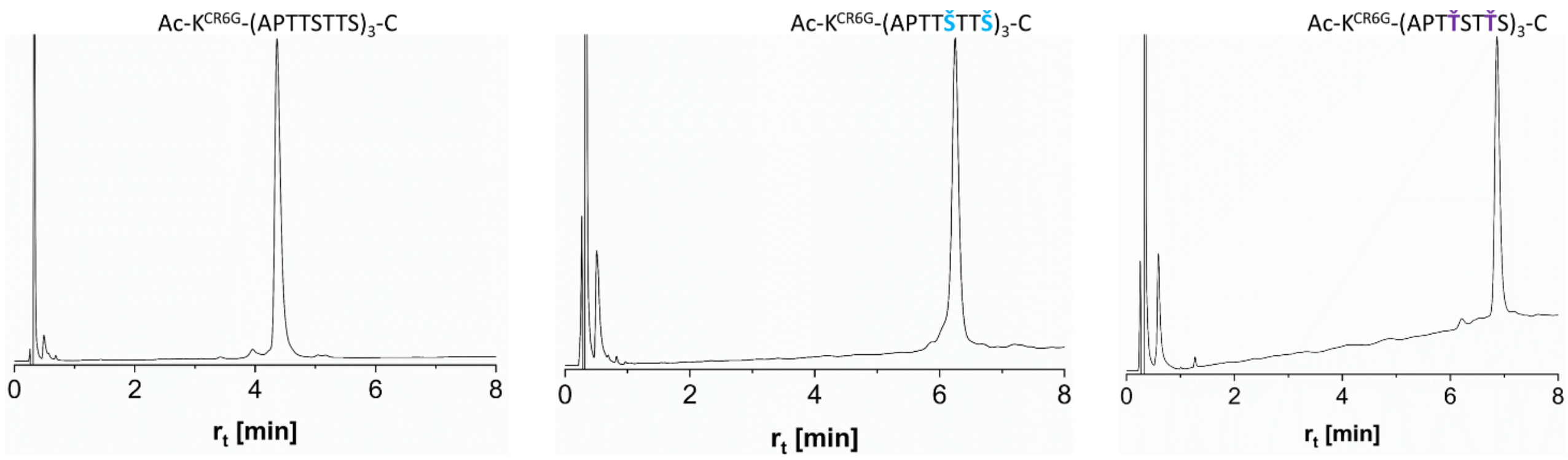

Figure S1. HPLC analysis of peptides **A-L1**, **B-L1**, and **C-L1**. Solvent A: 98.9% H_2O + 0.1% ACN + 0.1% TFA, solvent B: 98.9% ACN + 0.1% H_2O + 0.1% TFA. Gradient (A-L1): 20-25% B in A, Gradient (**B-L1**): 17-22% B in A, Gradient (**C-L1**): 17-22% B in A. λ = 210 nm.

Labeling with QSY7-C5 maleimide: After the peptides were purified by preparative HPLC, they were subjected to the labeling with QSY7-C5 maleimide.

The peptide was dissolved in the degassed buffer (25 mM TRIS, 150 mM NaCl, pH 7.08) to achieve 0.342 mM concentration. The QSY7-C5 maleimide was dissolved in dry DMF to prepare a stock solution of 10 mM concentration. Then, this stock solution was added to the peptide solution in TRIS buffer. The final concentration of the peptide was 0.2 mM and that of the maleimide 4.155 mM. The reaction mixture was incubated for 2 hours in a shaker at 25°C. Afterwards, it was diluted with water and lyophilized. Residual QSY7-C5 maleimide was removed via a desalting procedure employing Thermo Scientific Pierce™ peptide desalting spin columns. The eluates were lyophilized.

Finally, the peptides Ac-K^{CR6G}-(APTTSTTS)$_3$-C$_{QSY7}$ (**A-L2**), Ac-K^{CR6G}-(APTT**Š**TT**Š**)$_3$-C$_{QSY7}$ (**B-L2**), and Ac-K^{CR6G}-(APT**Ť**ST**Ť**S)$_3$-C$_{QSY7}$ (**C-L2**) were produced.

Ac-K^{CR6G}-(APTTSTTS)$_3$-C$_{QSY7}$ (**A-L2**)
Yield: 0.16 mg (41.2 nmol), quant. ESI-MS (pos. mode): m/z = 948.7 ($C_{176}H_{248}N_{35}O_{55}S_2$ $(M+4H)^{4+}$, calcd.: 948.9, 759.2 ($C_{176}H_{249}N_{35}O_{55}S_2$ $(M+5H)^{5+}$, calcd.: 759.3).

Ac-K^{CR6G}-(APTT**Š**TT**Š**)$_3$-C$_{QSY7}$ (**B-L2**)
Yield: 0.04 mg (7.66 nmol), quant. ESI-MS (pos. mode): m/z = 1253.3 ($C_{224}H_{326}N_{41}O_{85}S_2$ $(M+4H)^{4+}$, calcd.: 1253.5, 1002.9 ($C_{224}H_{327}N_{41}O_{85}S_2$ $(M+5H)^{5+}$, calcd.: 1003.0, 835.9 ($C_{224}H_{328}N_{41}O_{85}S_2$ $(M+6H)^{6+}$, calcd.: 836.0).

Ac-K^{CR6G}-(APT**Ť**ST**Ť**S)$_3$-C$_{QSY7}$ (**C-L2**)
Yield: 0.07 mg (13.13 nmol), quant. ESI-MS (pos. mode): m/z = 1253.3 ($C_{224}H_{326}N_{41}O_{85}S_2$ $(M+4H)^{4+}$, calcd.: 1253.5, 1002.9 ($C_{224}H_{327}N_{41}O_{85}S_2$ $(M+5H)^{5+}$, calcd.: 1003.0)

Mass Spectrometry

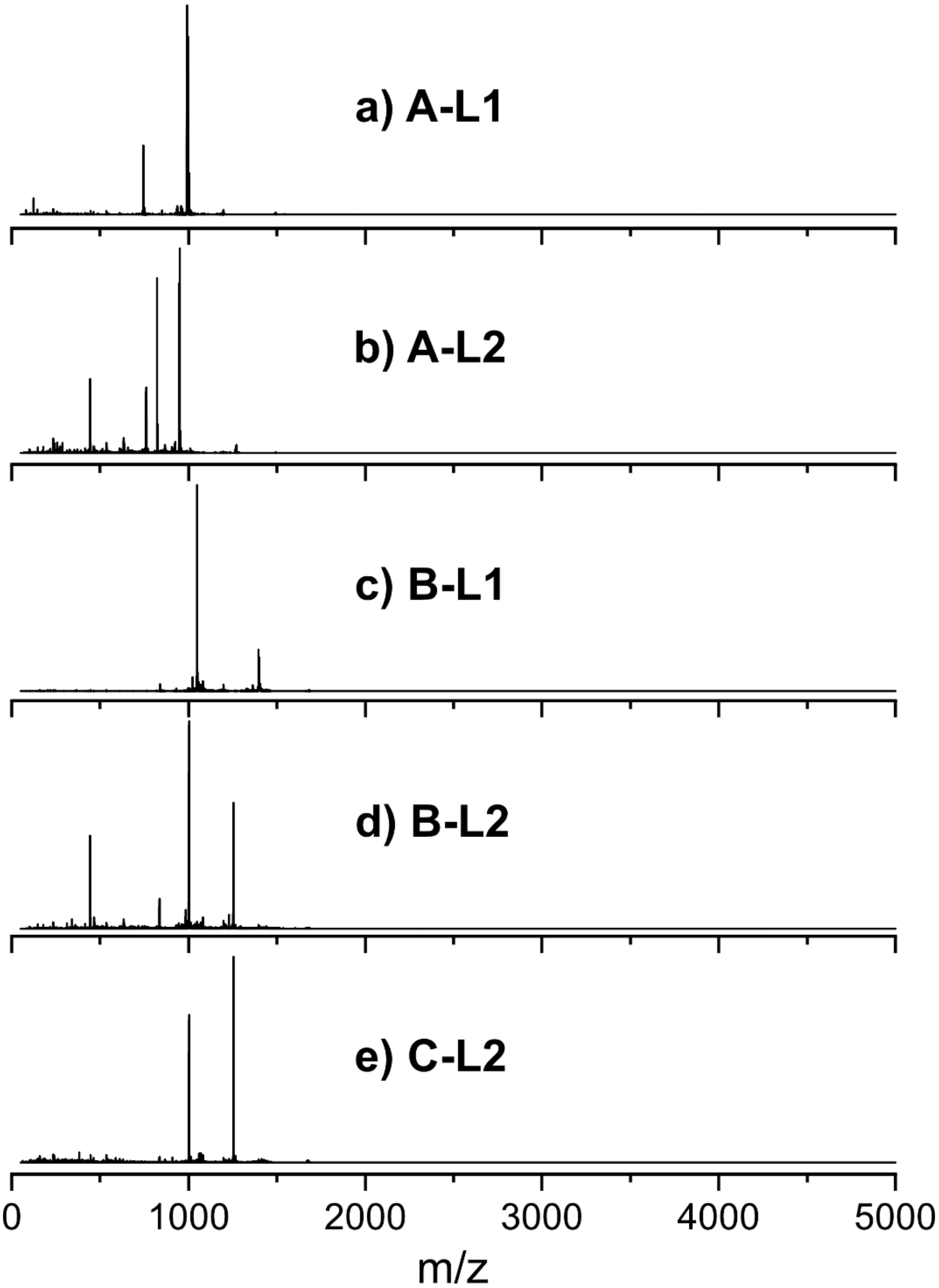

Figure S2. Full nESI mass spectra of (glyco)peptides shown in Figure 1. The (glyco)peptides (5 µM) are ionized from aqueous solutions containing 50 mM ammonium acetate.

Gas-Phase FRET

Using FRET, the distance between a fluorescence donor and a quencher/acceptor can be estimated by knowing the quenched and unquenched fluorescence lifetimes of singly- and doubly-labeled species (*e.g.*, peptides or proteins). From the fluorescence lifetimes, the FRET efficiency E can be derived (1).

$$E = 1 - \frac{t_{DA}}{t_D} \qquad (S1)$$

With the FRET efficiency, the donor-acceptor distances r_{DA} can be estimated with equation (2) if the Förster distance R_0 is known. R_0 is typically constant for an donor-acceptor pair and can be calculated using equation (3). While all parameters in equation (3) are known, for example, for the aqueous phase, they are not known for the gas phase. Therefore, gas-phase R_0 values have been approximated previously by using the value of R_0 in aqueous solution and exchanging the refractive index (n) of water, by that of the vacuum. It has been shown that gas-phase R_0 values obtained in this way might not be accurate,[3] and hence distances derived should at best be taken as a rough guideline. κ^2 is the orientation factor, QY is the quantum yield of the fluorescence donor, N_A is Avogadro's number, and $\int_0^\infty F_D(\lambda) \cdot \varepsilon_A(\lambda) \cdot \mathrm{d}\lambda$ describes the spectral overlap between the emission of the fluorophore and the absorption of the quencher.

$$r_{DA} = R_0 \cdot \sqrt[6]{\frac{1-E}{E}} \qquad (S2)$$

$$R_0 = \sqrt[6]{\frac{9000 \cdot \ln(10) \cdot \kappa^2 \cdot \mathrm{QY}}{128 \cdot \pi^5 \cdot N_A \cdot n^4} \cdot \int_0^\infty F_D(\lambda) \cdot \varepsilon_A(\lambda) \cdot \mathrm{d}\lambda} \qquad (S3)$$

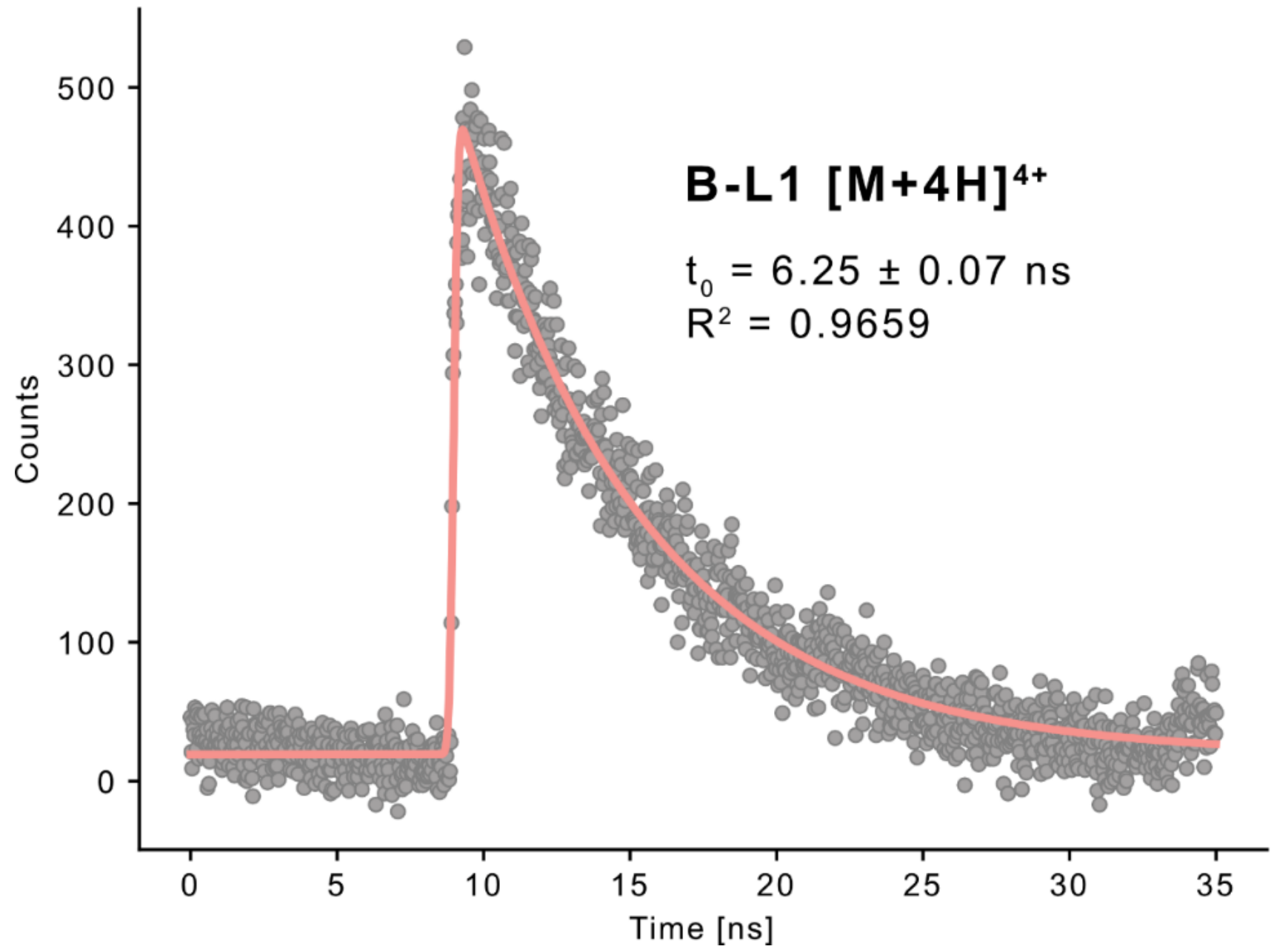

Figure S3. Gas-phase fluorescence decay curves of the glycopeptide **B-L1**, in the $[M+4H]^{4+}$ charge state. Ionized from 20 µM **B-L1** in 10 mM AA and excited with a 460 nm (10 mW) laser beam. The fluorescence lifetime is obtained from the fit (red).

Table S1. Gas-phase fluorescence lifetimes of (glyco)peptides and derived FRET efficiencies as well as donor-acceptor distances. The 5+ charge state of the peptides **A-L2** could not be generated in sufficient quantities to be measured with gas-phase FRET.

Molecule	Charge State	Lifetime [ns]	FRET Efficiency	Donor-Acceptor Distance$^{(a)}$
**A-L1**	$[M+3H]^{3+}$	6.28±0.05	-	-
**A-L2**	$[M+3H]^{4+}$	1.41±0.02	77.5±0.5%	58.7±0.3 Å
**B-L1**	$[M+3H]^{3+}$	6.96±0.05	-	-
	$[M+4H]^{4+}$	6.25±0.07	-	-
**B-L2**	$[M+3H]^{4+}$	0.53±0.01	92.4±0.2%	47.6±0.3 Å
	$[M+4H]^{5+}$	2.10±0.03	66.4±0.9%	64.5±0.5 Å
**C-L2**	$[M+3H]^{4+}$	0.48±0.01	93.1±0.2%$^{(b)}$	46.8±0.2 Å$^{(b)}$
	$[M+4H]^{5+}$	2.62±0.03	58.1±1.0%$^{(b)}$	68.4±0.5 Å$^{(b)}$

(a) Donor-Acceptor distances based on gas-phase R_0=72.2 Å. Gas-phase R_0 values cannot be accurately determined and derived distances should therefore be taken with caution, (b) these values are derived using the unquenched lifetime of **B-L1**.

Solution-Phase FRET

Table S2. Fluorescence lifetimes obtained by fitting experimental fluorescence decay curves from (glyco)peptides (250 nM) dissolved in pure H_2O (pH = 5.3–5.8), ammonium acetate (AA, 50 mM, pH = 6.8), or potassium phosphate buffer (PB, 100 mM, pH = 7.1). The glycopeptide solution was irradiated by a 480 nm laser beam with a power of 8–650 μW, adjusted based on the brightness of the peptide.

Fluorescence Lifetimes [ns]	Pure H_2O	50 mM AA	100 mM PB
**A-L1**	4.44 ± 0.02	4.44 ± 0.02	4.41 ± 0.02
**A-L2**	2.65 ± 0.01	2.65 ± 0.01	2.91 ± 0.01*
**B-L1**	4.45 ± 0.02	4.45 ± 0.02	4.43 ± 0.02
**B-L2**	3.45 ± 0.01	3.50 ± 0.01	3.42 ± 0.01
**C-L2**	4.24 ± 0.02	4.33 ± 0.02	4.25 ± 0.02

*The quenched lifetimes tend to increase with temperature/irradiation time.

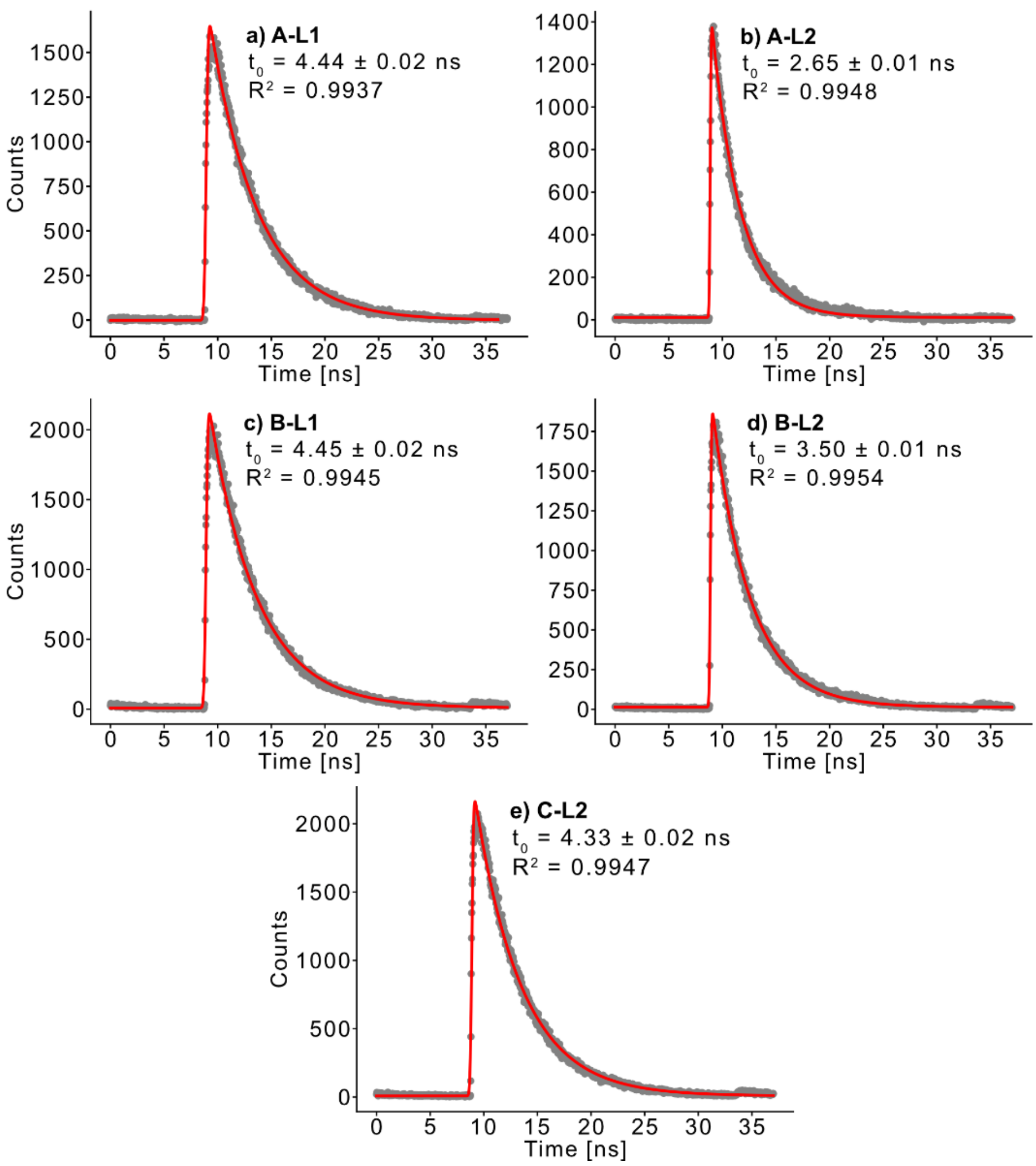

Figure S4. Solution-phase fluorescence decay curves of the (glyco)peptides (a) **A-L1**, (b) **A-L2**, (c) **B-L1**, (d) **B-L2**, and (e) **C-L2**. Recorded in 250 nM (glyco)peptide solutions (50 mM AA) at an excitation wavelength of 480 nm. The fluorescence lifetime is obtained from the fit (red).

Table S3. Solution-phase fluorescence lifetimes of (glyco)peptides and derived FRET efficiencies as well as donor-acceptor distances in 50 mM AA.

Molecule	Lifetime [ns]	FRET Efficiency	Donor-Acceptor Distance[(a)]
**A-L1**	4.44±0.02	-	-
**A-L2**	2.65±0.01	40.3±0.5%	63.7±0.3 Å
**B-L1**	4.45±0.02	-	-
**B-L2**	3.50±0.01	21.3±0.6%	74.2±0.6 Å
**C-L2**	4.33±0.02	2.7±0.9%	108.5±7.7 Å

(a) Donor-Acceptor distances based on aqueous phase R_0 = 59.7 Å, (b) these values are derived using the unquenched lifetime of **B-L1**.

Ion Mobility–Mass Spectrometry

Table S4. Average CCS values of the peptides determined by IM-MS at wave heights of 24 V, 25 V and 26 V. SD = standard deviation, RSD = relative standard deviation.

**Peptide**	**Charge State**	$^{TW}CCS_{N2}$ [A^2]					
		**24 V**	**25 V**	**26 V**	**Mean**	**SD**	**RSD [%]**
**A-L1**	**2^+**	/	608.2	/	608.2	/	/
	**3^+**	704.8	674.1	677.2	685.4	16.9	2.5%
	**4^+**	928.7	893.2	882.4	901.4	24.2	2.7%
**A-L2**	**2^+**	/	711.2	/	711.2	/	/
	**3^+**	788.5	762.9	759.9	770.4	15.7	2.0%
	**4^+**	1053.2	1025.4	1044.0	1040.8	14.2	1.4%
	**5^+**	/	1164.6	/	1164.6	/	/
**C-L2**	**2^+**	/	629.4	/	629.4	/	/
	**3^+**	873.1	838.1	834.2	848.4	21.4	2.5%
	**4^+**	1013.2	976.0	977.5	988.9	21.0	2.1%
	**5^+**	1282.6	1250.5	1245.3	1259.5	20.2	1.6%
**B-L1**	**2^+**	/	727.0	/	727.0	/	/
	**3^+**	778.4	752.3	748.6	759.8	16.3	2.1%
	**4^+**	960.9	934.6	933.7	943.1	15.5	1.6%
**B-L2**	**2^+**	/	496.4	/	496.4	/	/
	**3^+**	873.1	843.0	844.7	853.6	16.9	2.0%
	**4^+**	1000.0	976.0	977.5	984.5	13.5	1.4%
	**5^+**	1250.0	1233.2	1233.7	1239.0	9.6	0.8%

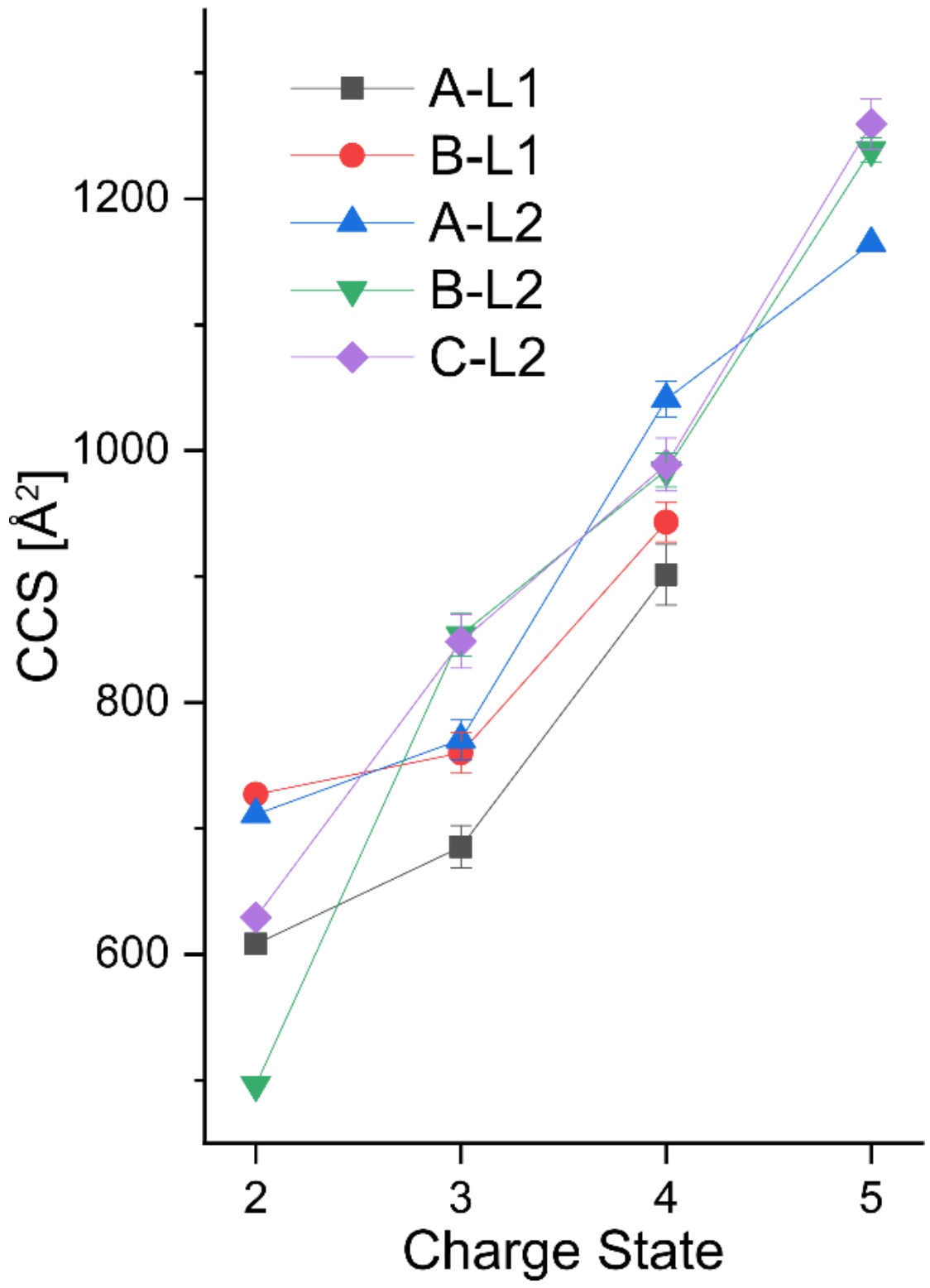

Figure S5. Collision cross sections of (glyco)peptides as a function of charge state. The results show that for the 4+ and 2+ charge states, the CCSs of the non-glycosylated peptide **A-L2** are larger than those of the corresponding hexaglycopeptides **B-L2** and **C-L2**.

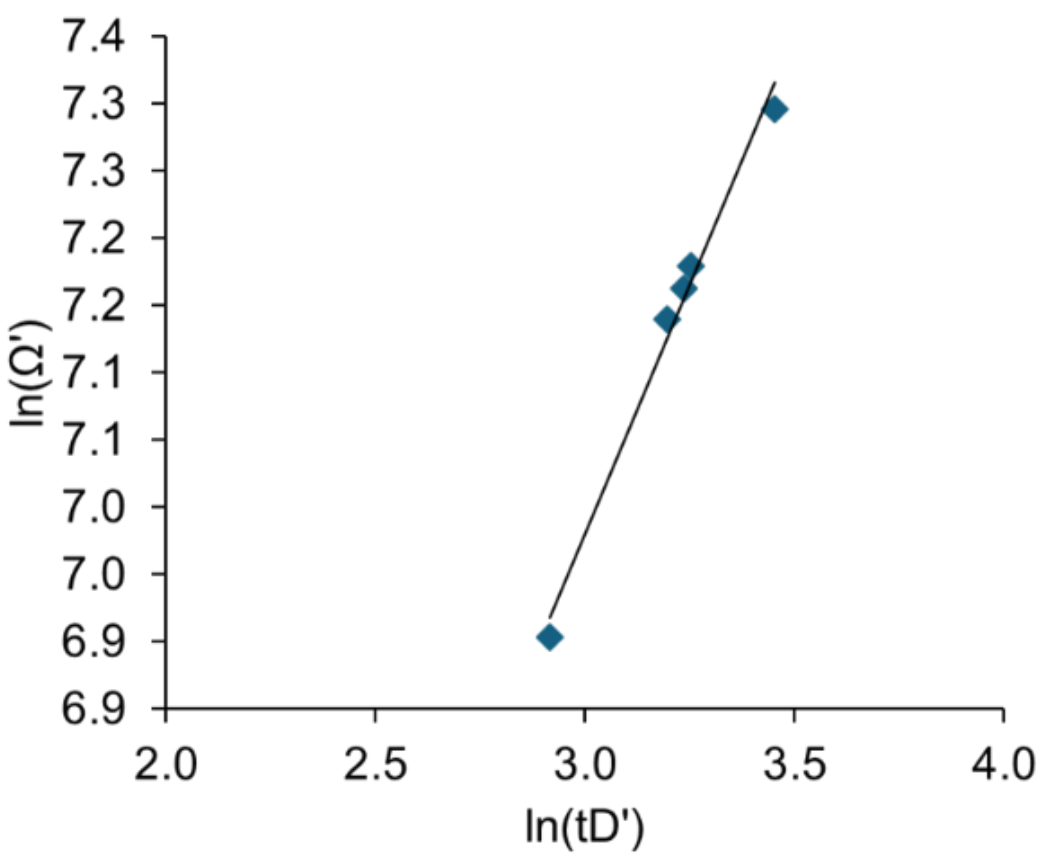

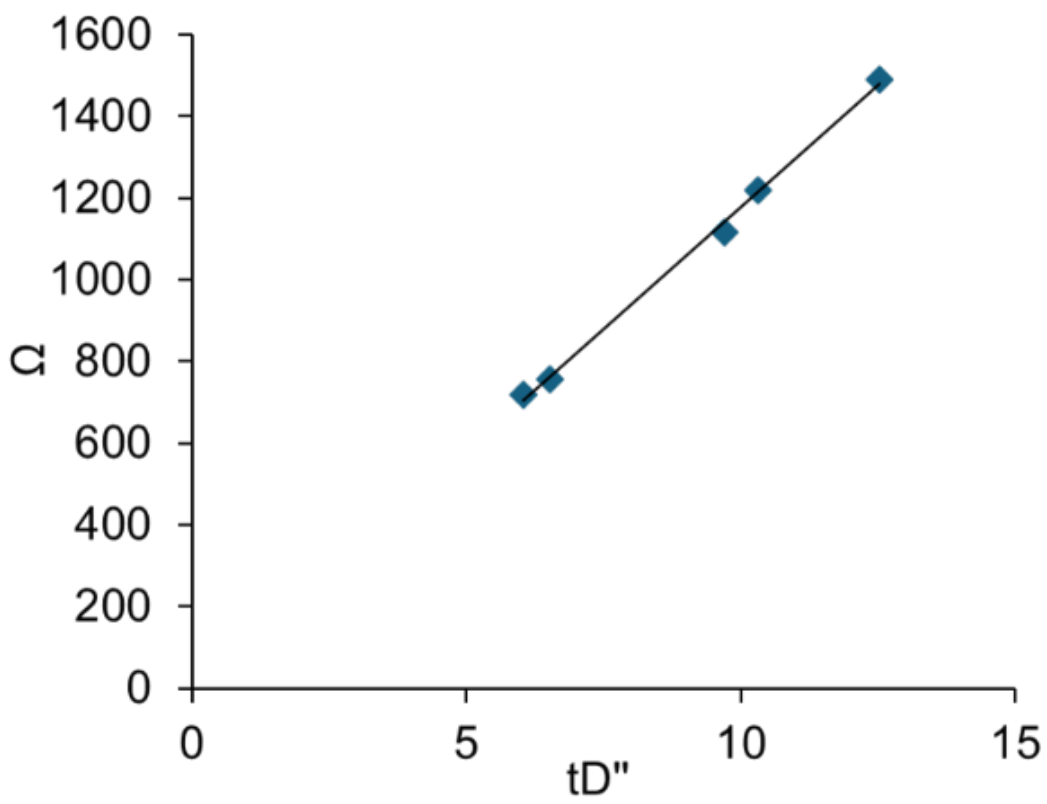

Figure S6. Calibration curves of the standards (melittin, ubiquitin and cytochrome c) at 25 V wave height (linear fit). The y-axis tick interval of the left figure is 0.05. Some adjacent tick labels therefore appear identical due to rounding to one decimal place.

Computational Results

Table S5. Computed CCSs for model (glyco)peptides APTTSTTS. The position of the posttranslational modification (a GalNAc residue) is denoted by a subscript G. As expected, the CCSs increase with increasing charge state and are higher with glycosylation than without glycosylation. To determine the expected increase in CCS, the CCSs of the non-glycosylated species are subtracted from those of the predicted singly-glycosylated sequences for a specific charge state and the same is done for doubly- vs. singly-glycosylated species. On average, glycosylation leads to an increase in CCS of 40±15 Å^2 per GalNAc residue. This would result in an approximate increase of 240 Å^2 when going from a non-glycosylated sequence to a hexaglycosylated sequence.

**CCS [Å^2]**	**APTTSTTS**	**APTT$_G$STTS**	**APTTS$_G$TTS**	**APTT$_G$STT$_G$S**	**APTTS$_G$TTS$_G$**
**1+**	262.8	298.9	306.5	342.5	355.5
**2+**	299.1	327	320.2	362.3	395.3
**3+**	355.4	376.3	404.7	426.7	432.7
**Difference**					
	-	36.1	43.7	43.6	49
	-	27.9	21.1	35.3	75.1
	-	20.9	49.3	50.4	28
	-			**Average**	**Standard Deviation**
	-			40.0	14.7

References

[1] A. Galashov, E. Kazakova, C. E. Stieger, C. P. R. Hackenberger, O. Seitz, *Chem. Sci.* **2024**, *15*, 1297–1305.

[2] A. Galashov, E. di Gregorio, P. Ponomareva, M. Safferthal, E. Kazakova, L. Bechtella, K. Pagel, M. Pigaleva, B. Joseph, O. Seitz, *Angew. Chem. Int. Ed.* **2025**, *64*, e202508278.

[3] A. U. Petersen, C. Kjaer, C. Jensen, M. Brondsted Nielsen, S. Brondsted Nielsen, *Angew. Chem. Int. Ed.* **2020**, *59*, 20946–20955.
